# Electroacupuncture negatively regulates the Nesfatin-1/ERK/CREB pathway to alleviate HPA axis hyperactivity and anxiety-like behaviors caused by surgical trauma

**DOI:** 10.1186/s13020-024-00974-2

**Published:** 2024-08-17

**Authors:** Jiayuan Zheng, Yu Wang, Chi Zhang, Anjing Zhang, Yuxiang Zhou, Yunhua Xu, Jin Yu, Zhanzhuang Tian

**Affiliations:** 1 Department of Integrative Medicine and Neurobiology, School of Basic Medical Sciences, State Key Laboratory of Medical Neurobiology and MOE Frontiers Center for Brain Science, Institutes of Brain Science, Institute of Acupuncture Research, Academy of Integrative Medicine, Shanghai Key Laboratory for Acupuncture Mechanism and Acupoint Function, Shanghai Medical College, Fudan University, Shanghai, 200032 China; 2 Department of Medical Oncology, Zhongshan Hospital, Fudan University, Shanghai, 200032 China; 3 Department of Rehabilitation Medicine, Huashan Hospital, Fudan University, Shanghai, 200040 China; 4 Department of Neurological Rehabilitation Medicine, The First Rehabilitation Hospital of Shanghai, Shanghai, 200090 China

**Keywords:** HPA axis, Anxiety, Nesfatin-1/ERK/CREB pathway, Surgical trauma, Electroacupuncture

## Abstract

**Background:**

Hyperactivity of the hypothalamic–pituitary–adrenal (HPA) axis constitutes a pivotal response by surgical trauma, manifesting as a critical aspect of the acute stress reaction. This hyperactivity resulted in adverse surgical outcomes and is often associated with increased postoperative anxiety. Increased evidence suggests that Nesfatin-1 plays a crucial role in stress responses and stress-related psychiatric disorders. Electroacupuncture (EA) is widely used to alleviate stress responses and anxiety, although its mechanism of action remains unclear. This study aimed to assess the mechanisms by which hypothalamic Nesfatin-1 contribute to the alleviation of HPA axis hyperactivity and anxiety by EA.

**Methods:**

Partial hepatectomy (HT) was performed to simulate surgical trauma, and EA was applied at Zusanli (ST36) and Sanyinjiao (SP6). The levels of hypothalamic Nesfatin-1, c-Fos, and corticotropin-releasing hormone (CRH) were detected, and serum adrenocorticotropic hormone (ACTH) and corticosterone (CORT) were regarded as indicators of HPA axis activity. Anxiety levels were assessed through open field tests (OFT), elevated plus maze (EPM), and light–dark box tests (LDBT). To investigate the role of Nesfatin-1, its expression was modulated using stereotactic viral injections or plasmid transfections. Transcriptome sequencing was employed to explore the downstream signaling pathways of Nesfatin-1. Additionally, brain cannula implantation was performed to facilitate targeted drug administration.

**Results:**

Our findings demonstrated that EA reduced the hypothalamic overexpression of CRH and Nesfatin-1, as well as serum levels of ACTH and CORT. Additionally, it alleviated anxiety-like behaviors resulting from surgical trauma. We observed that overexpression of Nesfatin-1 in the hypothalamic paraventricular nucleus (PVN) triggered hyperactivity of the HPA axis and anxiety. Conversely, knocking down Nesfatin-1 in the PVN reversed these effects caused by surgical trauma. Transcriptome sequencing identified the extracellular regulated protein kinases (ERK)/cAMP-response element binding protein (CREB) pathway as a key mediator in the impacts of surgical trauma and EA on the hypothalamus. Both in vivo and in vitro studies showed that overexpression of Nesfatin-1 activated the ERK/CREB pathway. Furthermore, administering ERK or CREB inhibitors into the PVN mitigated HPA axis hyperactivity and anxiety-like behaviors induced by surgical trauma. Finally, EA was observed to decrease the phosphorylation levels of ERK and CREB in the PVN.

**Conclusion:**

EA alleviates HPA axis hyperactivity and anxiety-like behaviors caused by surgical trauma through inhibition of Nesfatin-1/ERK/CREB pathway in the hypothalamus.

**Supplementary Information:**

The online version contains supplementary material available at 10.1186/s13020-024-00974-2.

## Introduction

Surgical procedures hold a crucial role in modern medicine, encompassing a wide array of applications such as the resection of cancerous tissues, repair of traumatic injuries, and treatment of heart diseases [1]. However, during the surgical process, patients are exposed to trauma, anesthesia, and pain, leading to the hyperactivity of the hypothalamic–pituitary–adrenal (HPA) axis. This represents a significant manifestation of the stress response triggered by surgical trauma [2]. Typically, the hyperactivity of the HPA axis is characterized by an increased synthesis and release of corticotropin-releasing hormone (CRH) in the hypothalamic paraventricular nucleus (PVN), along with elevated serum levels of adrenocorticotropic hormone (ACTH) and corticosterone (CORT) [3]. While the activation of the HPA axis is necessary for maintaining homeostasis, excessive hyperactivity of the HPA axis can result in severe and potentially life-threatening consequences [4]. These adverse effects include high metabolism, organ damage [5], systemic inflammatory responses, immune suppression [6], and psychological symptoms such as anxiety and delirium [1, 7]. Research indicates that patients experiencing postoperative anxiety often have prolonged hospital stays, lower postoperative satisfaction, poorer compliance with rehabilitation and treatment, and slower recovery rates [8]. Given the current lack of specific methods for the prevention and treatment of excessive stress responses following surgery and major trauma, exploring the mechanisms underlying their development and maintenance, as well as seeking solutions, holds significant clinical significance.

Acupuncture is one of the traditional Chinese therapeutic methods [9], and electroacupuncture (EA) represents a modern advancement of this practice. EA combines acupuncture stimulation with the subsequent electrophysiological effects, delivering pulsed stimulation with different waveforms to specific acupoints [10]. A substantial body of research suggests that acupuncture can improve perioperative complications, including intraoperative hemodynamic instability, immunosuppression [11], pain, nausea, vomiting, cognitive dysfunction, and anxiety [12–14]. Zusanli (ST36) and Sanyinjiao (SP6) are the most used acupoints for EA to alleviate hyperactivity of the HPA axis induced by acute stress [15]. Our previous studies also demonstrated that EA at these two acupoints can improve HPA axis hyperactivity in partial hepatectomy (HT) mice [16]. Although previous studies have indicated a significant alleviating effect of EA on the HPA axis hyperactivity and anxiety induced by surgical trauma [17–19], the molecular mechanisms underlying the therapeutic effects of EA still remain largely unknown.

Nesfatin-1 was initially discovered as a neuropeptide with anorexigenic effects, and as research progressed, its role in mediating stress and stress-related anxiety has been increasingly reported [20]. Nesfatin-1 is derived from nucleobindin2 (NUCB2) through cleavage [21], and it is massively distributed in the PVN [22]. It effects through binding to G protein-coupled receptors that have not yet been identified [23]. Studies have shown that administration of Nesfatin-1 into the lateral ventricle or intravenously in rats elevates serum ACTH and CORT levels [24, 25], which is associated with heightened anxiety [26]. Conversely, administration of Nesfatin-1 antibody or blockade of endogenous Nesfatin-1 centrally can attenuate its anxiety-like behaviors [27]. These findings indicate that central Nesfatin-1 is crucial for the activation of the HPA axis and the development of anxiety under stress.

In the present study, we observed that EA alleviates the hyperactivity of the HPA axis and anxiety resulting from surgical trauma by inhibiting the overexpression of Nesfatin-1 in the hypothalamus. Employing transcriptomic sequencing techniques, we further identified that the excessive secretion of CRH, induced by surgical trauma, is mediated by the Nesfatin-1/ extracellular regulated protein kinases (ERK)/cAMP-response element binding protein (CREB) pathway. Furthermore, EA demonstrates inhibitory effects on this pathway. This article innovatively investigates how EA ameliorates anxiety triggered by traumatic stress through the regulation of central Nesfatin-1 and its mediated function in the HPA axis. It offers a promising therapeutic strategy and identifies potential targets for treating endocrine and mental disorders associated with clinical surgery.

## Materials and methods

### Experimental animals

C57BL/6 J mice (male, 7–8 weeks, 20–23 g), were purchased from Yuxiu Biotechnology Co., Ltd. (Shanghai, China). All of them were housed in a room with a temperature of 22–24 °C, humidity ranging from 50 to 60%, and a 12-h light/dark cycle, with free access to food and water. After one week of acclimatization to the environment, experiments were conducted. All animal experiments were was reviewed and approved by the Ethics Committee for Laboratory Animals, School of Basic Medical Sciences, Fudan University (20240229–050).

### Partial hepatectomy model

The mice were divided into Intact, partial hepatectomy (HT), and HT + EA groups. All animals underwent 30 min of daily restraint adaptation for a period of 3 days. The surgical trauma model used in this experiment is as described earlier [17]. In brief, after intraperitoneal injection of 0.2 mL/10 g Avertin for anesthesia, a surgical incision was made in mice, approximately 3 cm long along the midline from the xiphoid process to the pubic symphysis. The abdominal cavity was fully opened, and 10% of the liver was resected from the left lobe. After removing 10% of the liver lobe, hemostasis was immediately achieved using a disinfected dry cotton ball for approximately 5 min. Finally, the abdominal cavity was sutured. Throughout the surgical procedure, environmental temperature and aseptic techniques were strictly controlled, and the surgery was conducted from 8:00 to 10:00 in the morning.

### EA

Mice in each group were habituated to the self-made fixing devices (50 mL centrifuge tubes with enough holes to make sure mice could breathe normally and facilitate EA stimulation) once a day for three consecutive days before the first EA. In the EA procedure, mice were safely restrained and kept awake, while the Intact and HT groups were also restrained but received no other interventions. The acupoints chosen for EA were “Zusanli” (ST36, located on the outer side below the knee joint, about 2 mm below the head of the fibula [28]) and “Sanyinjiao” (SP6, located 5 mm below the head of the fibula and 2 mm outside the anterior tibial tubercle) on the right hind limb. The EA occurred on the morning of the day before surgery and immediately after the abdominal closure [29]. The 0.5-inch (0.22 × 13 mm) acupuncture needles (Hua Tuo, Suzhou, China) were used, inserted vertically into the ST36 to a depth of approximately 5 mm, and inserted horizontally from bottom to top into the ST6 to a depth of about 5 mm. Both acupoints were connected to a HANS Acupoint Nerve Stimulator (LH202H, Beijing, China). The EA parameters were set as follows: dense-sparse wave, frequency of 2–15 Hz, intensity ranging from 1–2 mA, with slight tremors observed in the lower limbs as the endpoint, and a duration of 30 min. At the end of the experiment (24 h post-HT), animals in all groups were decapitated following administration of Avertin. Blood was then collected via retro-orbital bleeding, and the hypothalamus was harvested on ice.

### Enzyme linked immunosorbent assay

After peripheral blood collection via retro-orbital bleeding, centrifugation was conducted at 4 °C and 3000 rpm for 30 min. The upper serum layer was collected post-centrifugation. Enzyme-linked immunosorbent assay (ELISA) kits, purchased from Shanghai Lengton Biotechnology Co., Ltd. (Shanghai, China), were employed for the quantification of plasma ACTH and CORT levels.

### Real-time polymerase chain reaction

Total RNA was isolated from hypothalamic tissues using TRIzol Reagent (15596026, Life Technologies, USA). Subsequently, the RNA was reverse transcribed into cDNA using the PrimeScript RT Reagent Kit (RR036A, Takara, Japan) according to the manufacturer’s instructions. PCR was performed with the SYBR Premix Ex Taq kit (RR420B, Takara, Japan) and the QuantStudio 3 Real-Time PCR System (ThermoFisher, USA). The reaction volume consisted of 10 μL SYBR Premix Ex Taq mixture, 0.8 μL primer mixture, 2 μL cDNA template, and 6.8 μL ddH_2_O. Transcript levels were normalized to the GAPDH within the same sample. The mRNA primers were synthesized by Shanghai Sangon Biotech Co., Ltd. (Shanghai, China).

The primer sequences were as follows: NUCB2 (Forward: 5′AAG AAG TAG GAA GAC TGC GGA TGC3′; Reverse: 5′AGG ATT CTG GTG GTT CAG GTG TTC3′); CRH (Forward: 5′CTG TCG TCC TGC CTG CCT TG3′; Reverse: 5′TTC ACC CAT GCG GAT CAG AAC C3′); GAPDH (Forward: 5′AGA AGG TGG TGA AGC AGG CAT C3′; Reverse: 5′CGA AGG TGG AAG AGT GGG AGT TG3′). The relative mRNA levels were analyzed using the 2^^−ΔΔ^Ct method, normalized to GAPDH.

### Western blot

At 24 h post-surgery, hypothalamic tissues of mice were collected after the administration of Avertin. The hypothalamic tissues were lysed using RIPA lysis buffer (Biosharp, China) containing a mixture of proteinase and phosphatase inhibitors (Beyotime, China) and subjected to ultrasonic homogenization. The supernatant was centrifuged at 4 °C, 12,000 rpm for 20 min. Protein concentration was determined using a BCA assay kit (Beyotime, China). Equal amounts of protein were separated by 12% SDS-PAGE (ACE, China) and transferred onto PVDF membranes (Millipore, German). The membranes were blocked with TBST containing 5% skimmed milk powder or Rapid Protein-Free Blocking Buffer (ACE, China). Subsequently, the membranes were incubated overnight at 4 °C with primary antibodies against CRH (10944–1-AP, anti-rabbit, 1:1000, Proteintech), Nesfatin-1 (AF6895, anti-sheep, 1:1000, R&D), β-tubulin (10094–1-AP, anti-rabbit, 1:10000, Proteintech), phosphor-ERK1/2 (4370, anti-rabbit, 1:1000; Cell Signaling Technology), ERK1/2 (4695, anti-rabbit, 1:2000; Cell Signaling Technology), phosphor-CREB Ser133 (9198S, anti-rabbit, 1:1000; Cell Signaling Technology), and CREB (12208–1-AP, anti-rabbit, 1:1000; Proteintech). Following washes with TBST, the membranes were incubated with secondary antibodies, either HRP-conjugated goat anti-rabbit (L-3012, 1:10000; SAB) or rabbit anti-sheep (AS023, 1:10000; ABclonal). Signal visualization was performed using ECL (Epizyme, China), and protein bands were detected using the Amersham ImageQuant 800 Protein Blot Imaging System. ImageJ software was employed for quantifying the grayscale values of the bands, and the relative expression of the target proteins was calculated based on the grayscale values of internal reference bands.

### Open field tests

The anxiety levels of mice were assessed using the open field tests (OFT) 24 h after the surgery [30]. The apparatus consisted of a square arena with opaque plastic walls (length = width = 50 cm, height = 40 cm). Each mouse was gently placed in the center of the arena, and allowed to explore the area for 10 min, during which their movement trajectory was recorded. Parameters evaluated included time spent in the central zone, central zone cross counts, the ratio of central distance to total distance, and grooming episode. After each trial, residual odors were eliminated using 75% ethanol.

### Elevated plus maze

Elevated plus maze (EPM) apparatus consists of two enclosed arms (length 20 cm, width 4 cm, height 12 cm) and two similar open arms arranged in a cross. A 10-min test is employed to determine the time spent in the open arms and entries made into the open arms. The apparatus is cleaned with 75% ethanol to eliminate residual odors left by the preceding animal.

### Light–dark box tests

The apparatus used for this experiment consisted of a box (35 × 25 × 30 cm) divided into two compartments: one-third of the box (dark) and two-thirds of the box (light). Mice were placed in the light compartment and allowed to freely explore the room for 10 min. A video camera was utilized to record the mouse's movements in the light area. Anxiety-like behaviors in mice were assessed based on the time spent and the distance traveled in the light area. The apparatus was cleaned with 75% ethanol to eliminate residual odors left by the preceding animal.

### Immunofluorescence

Mouse coronal brain slices (thickness 40 μm) were obtained, and selected slices were subjected to immunofluorescent staining. In brief, brain samples were incubated or co-incubated with antibodies against c-Fos (226008, anti-rabbit, 1:400, SYSY), Nesfatin-1 (AF6895, anti-sheep, 1:200, R&D), CRH (C36806, anti-rabbit, 1:200, SAB), or phosphor-ERK1/2 (4370, anti-rabbit, 1:200, Cell Signaling) at 4 °C overnight. The slices were then incubated with the respective secondary antibodies, Alexa Fluor 488 (A-21206, 1:1000, ThermoFisher) or Alexa Fluor 594 (A-11016, 1:1000, ThermoFisher), at room temperature in the dark for 2 h. Visualization was conducted using the integrated fluorescence microscopy system BZ-X (KEYENCE, Japan). For quantitative analysis of immunostained cells, ImageJ software was used to count the numbers of CRH, Nesfatin-1, c-Fos, phosphor-ERK1/2 positive cells, and co-labeled cells within a 400 µm^2^ area adjacent to the third ventricle (3 V).

### Virus injection

After intraperitoneal injection of Avertin for anesthesia, mice were placed in a stereotaxic apparatus. Following fixation, the hair on the head was shaved, and the surgical site was treated with a dilute iodine solution. An incision was made in the scalp, and the surgical area was swabbed with a surgical sponge soaked in hydrogen peroxide until the skull was exposed. The location of the PVN of the hypothalamus was (AP 0.6 mm, ML ± 0.2 mm) [29, 31]. Using a dental drill, a hole was slowly drilled into the skull, and a Hamilton 2.5 μL microsyringe, coupled with a glass electrode, was used to extract the virus. The viruses used were rAAV2/9-CMV-Nesfatin-1-EGFP-WPRE-hGH or rAAV2/9-U6-CMV-shRNA (scramble)-mCherry-SV40 obtained from BrainVT A Co., Ltd. (Wuhan, China). The virus was slowly inserted into the PVN to a depth of 4.5 mm at a rate of 40 nL/min, and a total injection volume of 200 nL. After the injection was completed, a 5-min waiting period was observed to prevent viral overflow. Subsequently, the skin on the head was sutured.

### Cell culture and plasmid transfection

The mouse neuroblastoma-2a (N2a) cells were obtained from the Center for Excellence in Molecular Cell Science, Chinese Academy of Sciences, and cultured in DMEM (Biosharp, China) supplemented with 10% fetal bovine serum, 50 units/mL penicillin, and 100 µg/mL streptomycin (Biosharp, China) at 37 °C in a humidified atmosphere with 5% CO_2_.

Overexpression or knockdown of Nesfatin-1 in N2a cells was achieved through plasmid transfection. The plasmid backbone is consistent with the viral nucleic acid framework. For transfection, N2a cells were plated at 60% density in 6-well culture plates, and JetPrime transfection reagent (PolyPlus Transfection, France) was used. mRNA or protein was extracted 48 h post-transfection, with a transfection efficiency of approximately 75%. In interventions with inhibitors, SCH772984 (HY-50846, MCE) or 666–15 (HY-101120, MCE) was added 24 h after plasmid transfection, following the same steps as before.

### Transcriptome sequencing

Sprague–Dawley (SD) rats (male, 7–8 weeks, 180–220 g) were purchased from the Slack Laboratory Animal Center (Shanghai Branch of the Chinese Academy of Sciences, Shanghai, China). Rats were in good health without any pre-existing conditions that could affect the study results. They successfully adapted to the experimental environment for one week before the experiment. Rats were randomly assigned to the three groups (Intact, HT, EA) using a random number generator. To ensure objectivity and reduce bias, all experimental assessments and subsequent analyses were conducted by researchers blinded to the group allocations. The groups were coded, and the code was not revealed until the completion of the data analysis. The samples were prepared using the Seq-RNA sequencing method provided by Novo Gene Biotech Co. Ltd. (Beijing, China). Three SD rats from each group were included. The surgical and EA procedures were identical to those performed in mice. 24 h after HT, the hypothalamic tissues were isolated from the rats, preserved in RNA storage solution, and sent to Novo Gene Biotech Company for subsequent analysis. The construction of cDNA libraries, library purification, and transcriptome sequencing were conducted following the protocols provided by Novo Gene Biotech Company. The normalized RNA count data was used for subsequent Principal Component Analysis (PCA) in R. Differential Expression analysis for RNA-Seq data was performed using R/Bioconductor package edgeR. The threshold of significance was set as a p value < 0.05 to find transcriptionally regulated genes. Heatmaps were made using R/Bioconductor package pheatmap (https://CRAN.R-project.org/package=pheatmap). Volcano plots were made using ggplot2 in R.

### Stereotactic cannula implantation and PVN administration

The bilateral PVN cannulation in mice was performed under Avertin anesthesia. The drug delivery system was designed by RWD Life Science Co., Ltd. (RWD, Shenzhen, China). Modeling and drug administration were carried out two weeks after the cannulation. On the day before surgery and immediately after surgery, mice were administered ERK1/2 inhibitor SCH772984 (0.1 nmol/µL, 0.5 µL/side, HY-50846, MCE), CREB inhibitor 666–15 (0.1 nmol/µL, 0.5 µL/side, HY-101120, MCE), or normal saline (NS) at a rate of 0.1 µL/min into the PVN [29]. After injection, the mice were left undisturbed for 5 min to allow for drug diffusion. All mice were euthanized under anesthesia 24 h after the last administration. Peripheral blood and hypothalamic tissue were collected and frozen for further experiments.

### Statistical analysis

All data were analyzed using GraphPad prism 9.0 software. Data were presented as mean ± SEM, and comparisons between two groups were performed using unpaired two-tailed t-tests. Multiple group comparisons were conducted using one-way ANOVA or two-way ANOVA. A p-value < 0.05 was considered statistically significant.

## Results

### EA alleviates the HPA axis hyperactivity and anxiety-like behaviors caused by surgical trauma

**Fig. 1:**
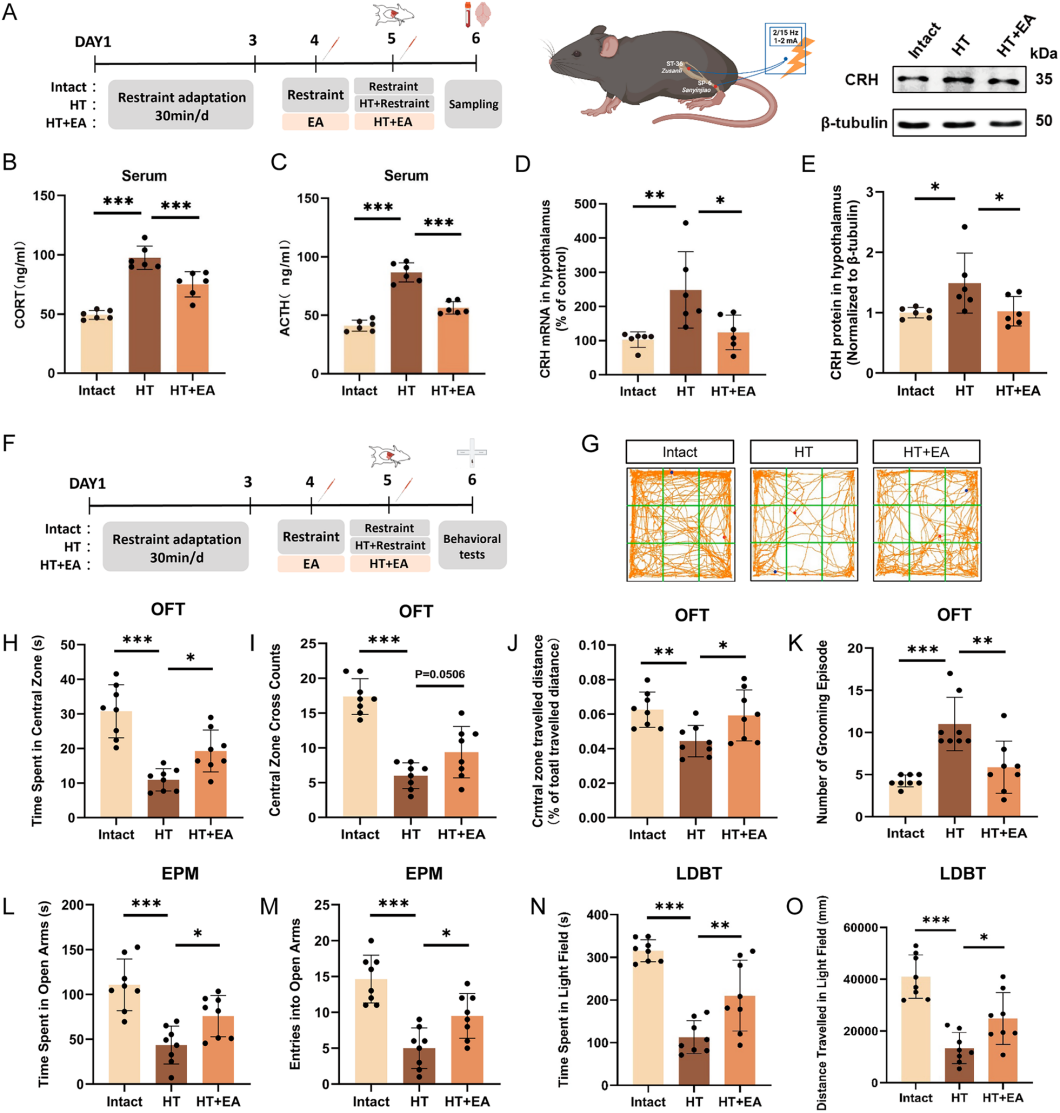
EA alleviates surgery-induced HPA axis hyperactivity and anxiety. **A**, **F** Schematic representation of the HT model and EA protocol. **B**, **C** ELISA measurement of serum ACTH and CORT levels. **D**, **E** Quantification of CRH mRNA and protein in hypothalamus. Anxiety levels in mice post-surgery were assessed through the OFT (**G**–**K**), the EPM (**L**, **M**) and the LDBT (**N**, **O**). All data are shown as mean ± SEM, n = 6–8 in each group, * p < 0.05, ** p < 0.01, ***p < 0.001

**Fig. 2:**
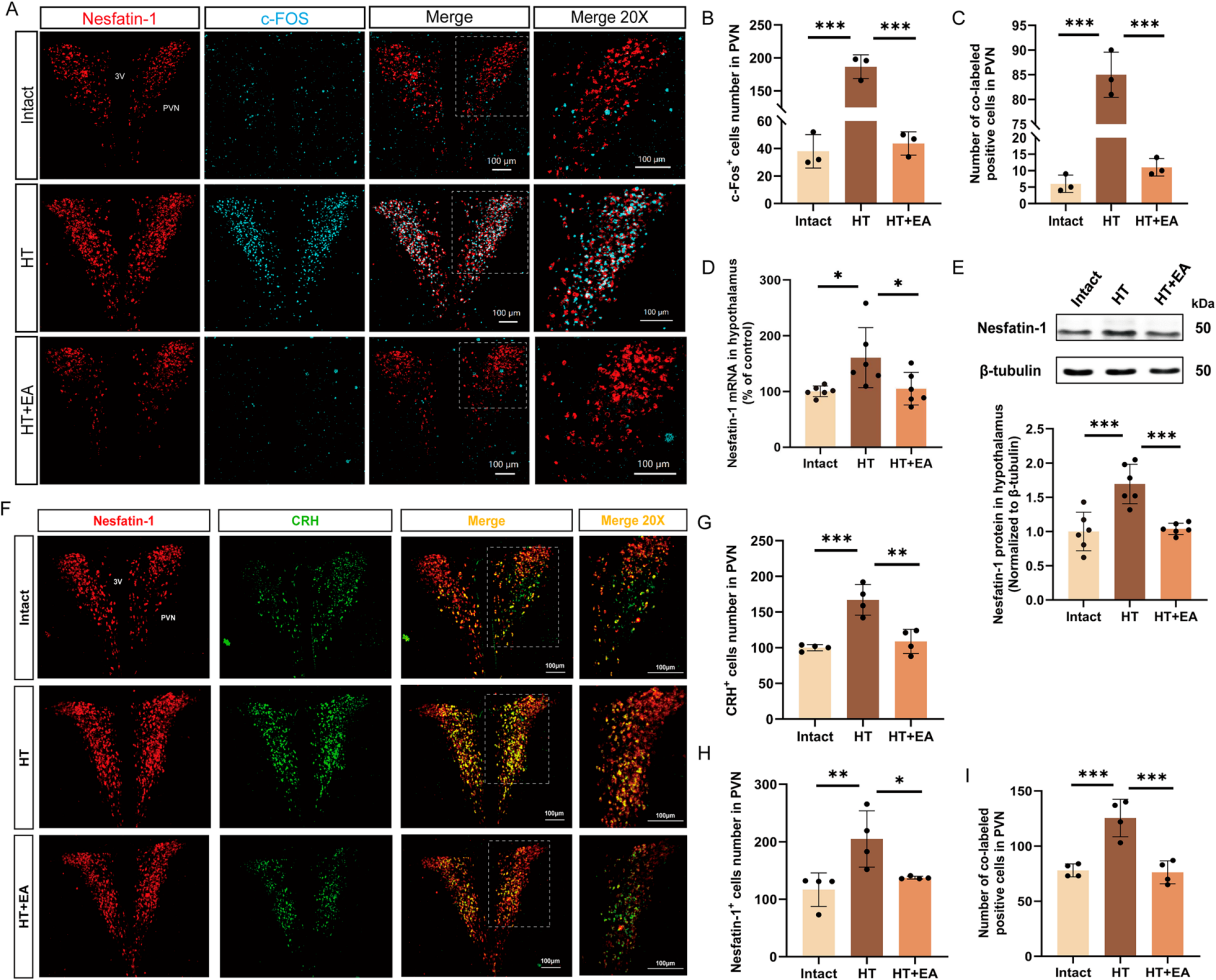
EA alleviates surgical trauma-induced overexpression of Nesfatin-1 in the hypothalamus. **A** Immunofluorescence staining for Nesfatin-1 and c-Fos in the PVN at 24 h post-surgery in the Intact, HT, and EA + HT groups. Scale bar = 100 μm. **B**, **C** Quantification of c-Fos-positive cells and their co-labeled with Nesfatin-1 in each group at 24 h post-surgery. **D**, **E** Expression levels of Nesfatin-1 mRNA and protein in the hypothalamus in each group. **F** Immunofluorescence staining for Nesfatin-1 and CRH in the PVN at 24 h post-surgery. Scale bar = 100 μm. **G**–**I** Quantification of CRH-positive cells, Nesfatin-1-positive cells, and their co-labeled cells in each group. All data are shown as mean ± SEM, n = 3–6 in each group, * p < 0.05, ** p < 0.01, ***p < 0.001

The experimental design and workflow are depicted in Fig. 1A, F. We assessed serum concentrations of ACTH and CORT, as well as CRH expression in the hypothalamus 24 h post-surgery to evaluate HPA axis activity. Anxiety-like behaviors were evaluated by the OFT, EPM, and light–dark box tests (LDBT). Compared to the Intact mice, the HT mice exhibited a significant increase in plasma levels of ACTH and CORT, as well as an increase in CRH mRNA and protein expression in the hypothalamus, and a higher count of CRH-positive cells in the PVN post-surgery. Interestingly, the combination of preoperative and postoperative EA significantly mitigated surgical trauma-induced hyperactivity of the HPA axis, as evidenced by substantial reductions in the expression levels of various HPA axis components (Fig. 1B–E, Fig. 2G).

The OFT results demonstrated that EA significantly reversed the decreased time spent in the central zone, reduced traveled distance in the central zone (normalized to total distance), decreased crossing counts of the central zone, and increased grooming episodes observed in HT mice (Fig. 1G–K). The EPM results indicated that EA significantly reversed the decreased time spent in the open arms and reduced entries into the open arms observed in HT mice (Fig. 1L, M). The LDBT results showed that EA significantly reversed the decreased time spent and traveled distance in the light area observed in HT mice (Fig. 1N, O). These results indicate that EA could alleviate the hyperactivity of the HPA axis caused by surgical trauma and further improve anxiety-like behaviors.

### EA mitigates overexpression of hypothalamic Nesfatin-1 induced by surgical trauma

Given the crucial role of Nesfatin-1 in stress and stress-induced anxiety, our investigation concentrated on this peptide within the hypothalamus, exploring it as a potential target for EA intervention. Firstly, we performed co-staining of Nesfatin-1 with c-Fos. At 24 h post-surgery, the HT mice showed elevated numbers of c-Fos-positive cells and their co-labeled with Nesfatin-1 in the PVN compared to the Intact mice. There was a significant reduction in the c-Fos expression and their co-labeled with Nesfatin-1 within PVN following EA. (Fig. 2A–C). In comparison to the Intact mice, the HT mice exhibited significant upregulation of Nesfatin-1 mRNA and protein levels in the hypothalamus, increased numbers of Nesfatin-1-positive cells, and their co-labeled with CRH in the PVN. These alterations were also effectively inhibited by EA (Fig. 2D–F, H, I). These data confirm that EA could directly regulate the overexpression of Nesfatin-1 induced by surgical trauma.

### Knockdown of Nesfatin-1 expression in the PVN alleviates surgical trauma-induced HPA axis hyperactivity and anxiety-like behaviors

**Fig. 3:**
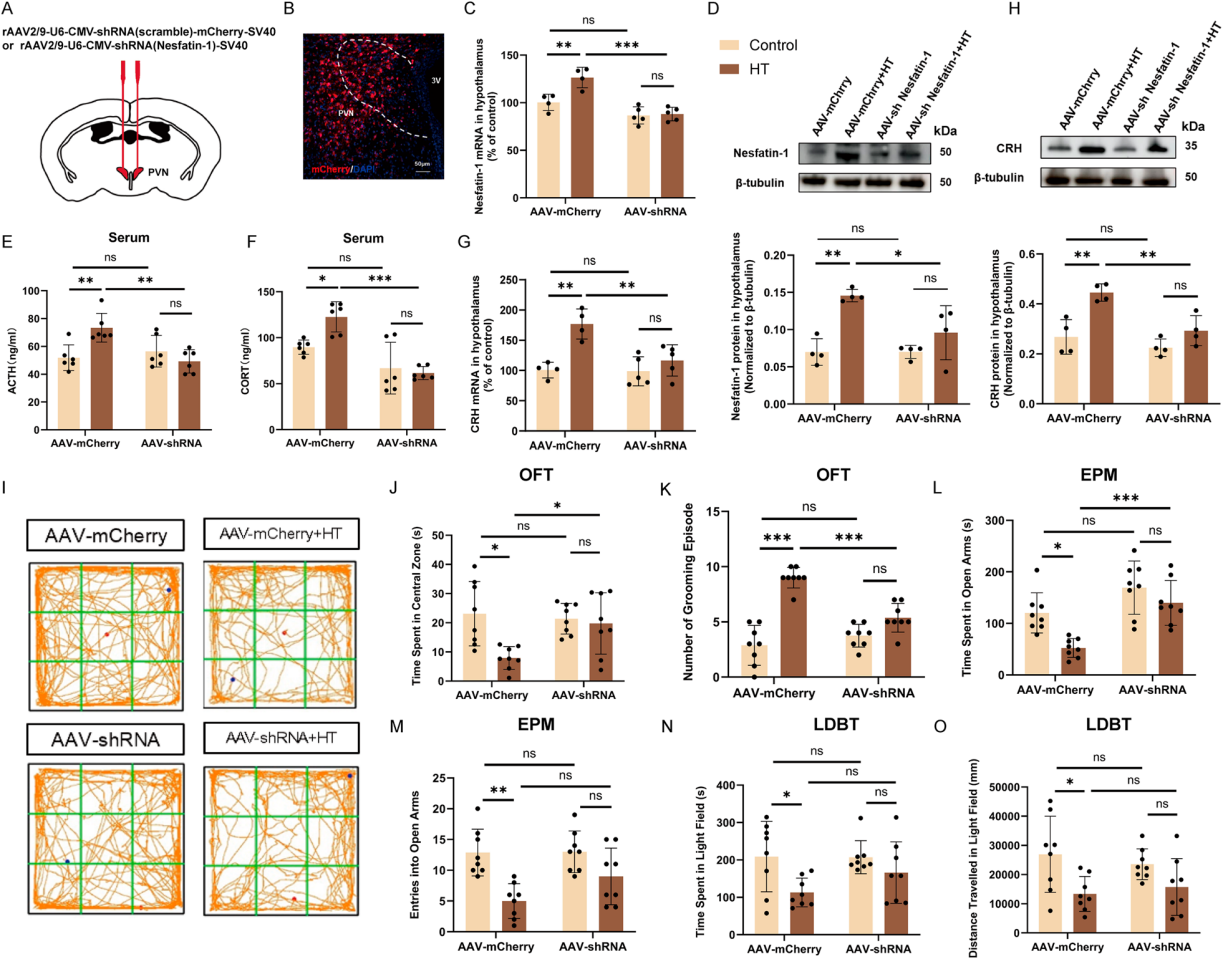
Knockdown of Nesfatin-1 in PVN alleviates HPA axis hyperactivity and anxiety-like behaviors induced by surgical trauma. **A**, **B** Diagram illustrating the sites of virus injection. Scale bar = 50 μm. **C**, **D** Expression levels of Nesfatin-1 mRNA and protein in the hypothalamus from different groups. **E**, **F** Serum levels of ACTH and CORT in different groups. **G**, **H** Expression levels of CRH mRNA and protein in the hypothalamus from different groups. OFT (**I**–**K**), EPM (**L**, **M**), and LDBT (**N**, **O**) were used to assess anxiety levels in different groups of mice. All data are shown as mean ± SEM, n = 4–8 in each group, * p < 0.05, ** p < 0.01, ***p < 0.001

To further investigate the role of Nesfatin-1 in the adverse consequences of surgical trauma, we administered AAV-shRNA targeting Nesfatin-1 into the PVN to achieve Nesfatin-1 knockdown (Fig. 3A). Three weeks post-virus injection, the area of virus infection was visualized (Fig. 3B), following which the mice were subjected to HT. In mice injected with AAV-mCherry (scramble), there were significant increases in the hypothalamic Nesfatin-1 mRNA and protein, CRH mRNA and protein, and serum levels of ACTH and CORT in the HT mice compared to the control mice (Fig. 3C–H). In mice injected with AAV-shRNA (Nesfatin-1), no significant differences were observed in these parameters when HT mice compared to the control mice (Fig. 3C–H).

The OFT results showed that HT mice injected with scramble virus exhibited a significant decrease in time spent in central zone (Fig. 3I, J), while the grooming episodes significantly increased (Fig. 3K). The EPM results showed that HT mice injected with scramble virus exhibited a significant decrease in the time spent in the open arms and entries into the open arms (Fig. 3L, M). The LDBT results showed that HT mice injected with scramble virus exhibited a significant decrease in the time spent and distance traveled in the light area (Fig. 3N, O). However, when PVN Nesfatin-1 is knocked down, the aforementioned anxiety behaviors are alleviated (Fig. 3I–O). These results indicate that knockdown of Nesfatin-1 in the PVN could alleviate HPA axis hyperactivity and further anxiety-like behaviors caused by surgical trauma.

### Overexpression of Nesfatin-1 in the PVN induces the HPA axis hyperactivity and anxiety-like behaviors

**Fig. 4:**
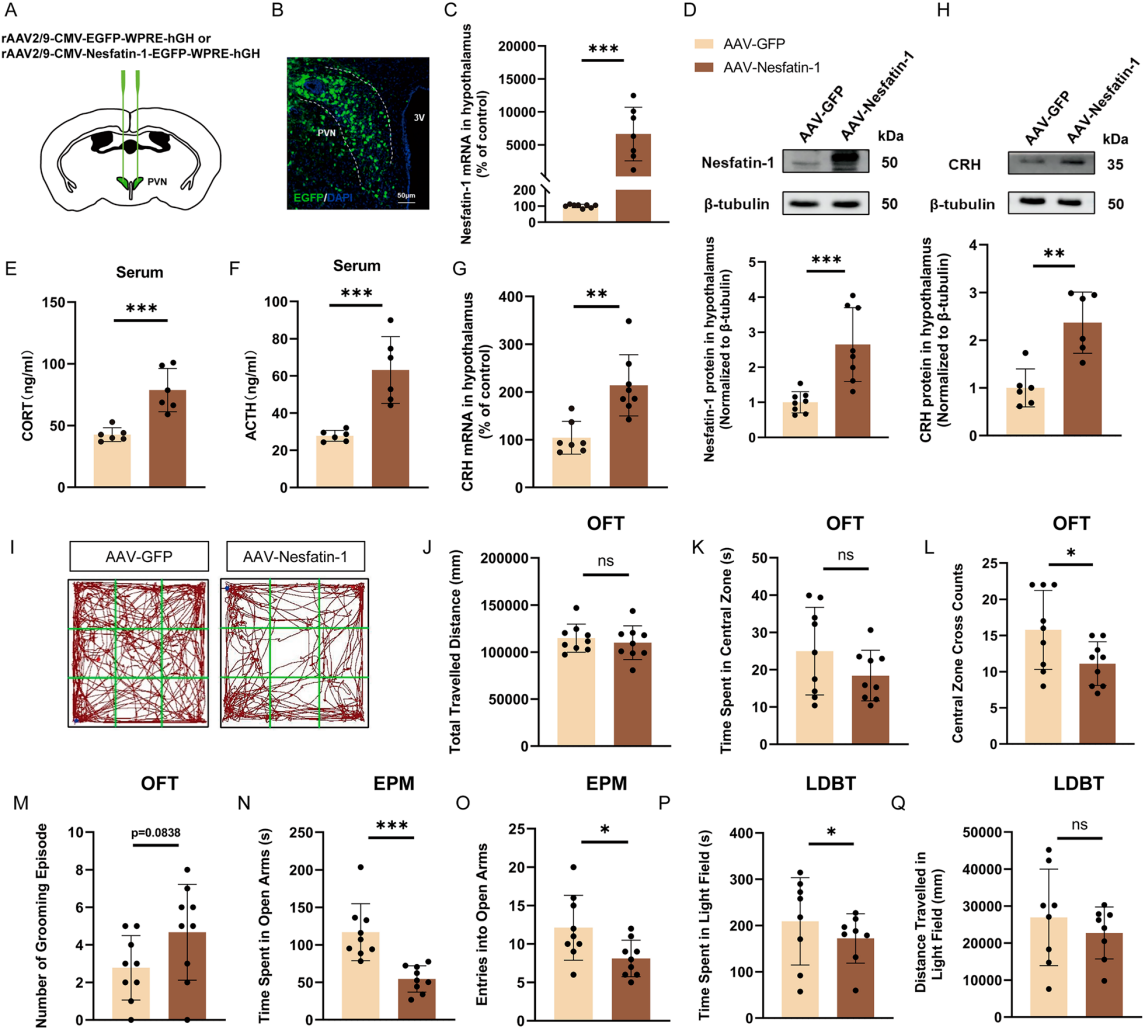
PVN overexpression of Nesfatin-1 induces HPA axis hyperactivity and anxiety-like behaviors. **A**, **B** Diagram of virus injection sites. Scale bar = 50 μm. **C**, **D** Expression levels of Nesfatin-1 mRNA and protein in the hypothalamus in each group. **E**, **F** Serum levels of ACTH and CORT in each group. **G**, **H** Expression levels of CRH mRNA and protein in the hypothalamus in each group. OFT (**I**–**M**), EPM (**N**, **O**), LDBT (**P**, **Q**, each group had one animal death) were used to assess anxiety levels in mice in each group. All data are shown as mean ± SEM, n = 6–9 in each group, * p < 0.05, ** p < 0.01, ***p < 0.001

AAV-Nesfatin-1 was injected into the PVN to achieve overexpression of Nesfatin-1 (Fig. 4A). Three weeks post-virus injection, the infected area was observed (Fig. 4B). Compared to the AAV-GFP (scramble) group, mice in the AAV-Nesfatin-1 group showed a significant increase in Nesfatin-1 mRNA and protein levels in the hypothalamus (Fig. 4C, D), indicating successful overexpression of Nesfatin-1. Nesfatin-1 overexpression significantly elevated plasma ACTH and CORT levels, as well as hypothalamic CRH mRNA and protein levels (Fig. 4E–H). This suggests a positive regulatory effect of Nesfatin-1 on the HPA axis.

In the OFT, AAV-Nesfatin-1 mice showed a significant decrease in central zone cross counts compared to the AAV-GFP group (Fig. 4L), while total travelled distance, time spent in central zone and grooming episodes showed no significant difference (Fig. 4I–K, M). In the EPM, AAV-Nesfatin-1 mice exhibited a significant decrease in time spent on and entries into the open arms (Fig. 4N, O). LDBT results indicated that AAV-Nesfatin-1 mice spent significantly less time in the light zone compared to the AAV-GFP group (Fig. 4P), with no significant differences in distance travelled in light zone (Fig. 4Q). These results suggest that overexpression of Nesfatin-1 in the PVN can induce HPA axis hyperactivity and anxiety-like behaviors.

### Nesfatin-1 regulates the expression of CRH

**Fig. 5:**
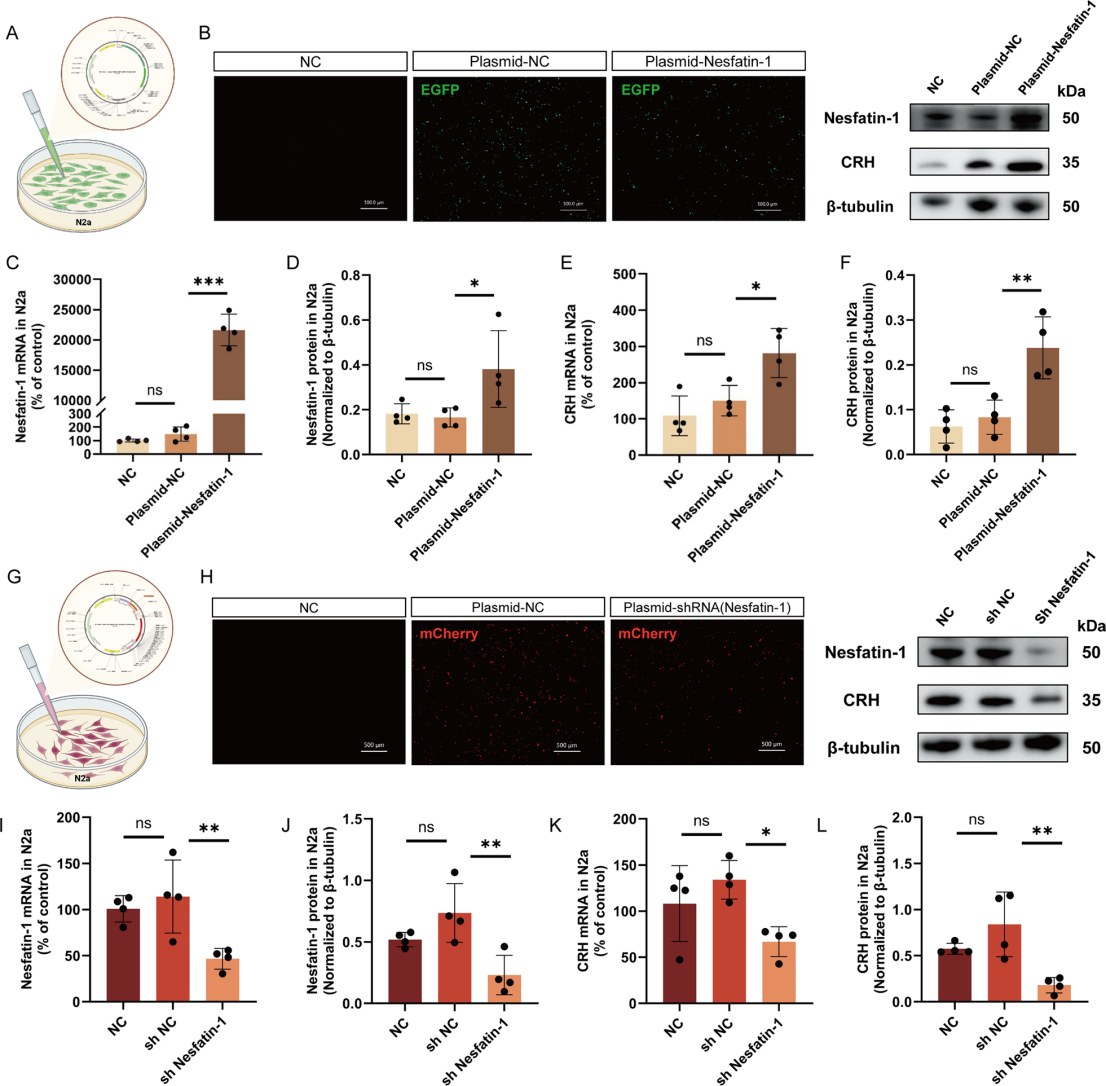
Regulation of CRH expression by Nesfatin-1. **A**, **G** Schematic representation of plasmid transfection. **B**, **H** Fluorescent images of N2a cells after 48 h of plasmid transfection. Scale bar = 500 μm. **C**, **D, I**, **J** Expression levels of Nesfatin-1 mRNA and protein in cells from each group. **E**, **F**, **K**, **L** Expression levels of CRH mRNA and protein in cells from each group. All data are shown as mean ± SEM, n = 4 in each group, * p < 0.05, ** p < 0.01, ***p < 0.001

To further explore the molecular mechanism through which HT induces HPA axis dysfunction through Nesfatin-1, we investigated the regulatory effect of Nesfatin-1 on CRH, the initiating factor of the HPA axis. Here, we achieved overexpression or knockdown of Nesfatin-1 in N2a cells through transfection (Fig. 5A, G). After 48 h of transfection, we observed the fluorescence in the cells (Fig. 5B, H) and measured the mRNA and protein levels of Nesfatin-1 and CRH. In cells transfected with the plasmid overexpressing Nesfatin-1, we found a significant increase in Nesfatin-1 and CRH mRNA and protein levels compared to the Plasmid-NC group (Fig. 5C–F). Similarly, in cells transfected with the shRNA targeting Nesfatin-1, we observed a significant decrease in Nesfatin-1 and CRH mRNA and protein levels compared to the Plasmid-NC group (Fig. 5I–L). These results suggest that Nesfatin-1 positively regulate the expression of CRH.

### Surgical trauma and subsequent EA induce alterations in the hypothalamic transcriptional profile

**Fig. 6:**
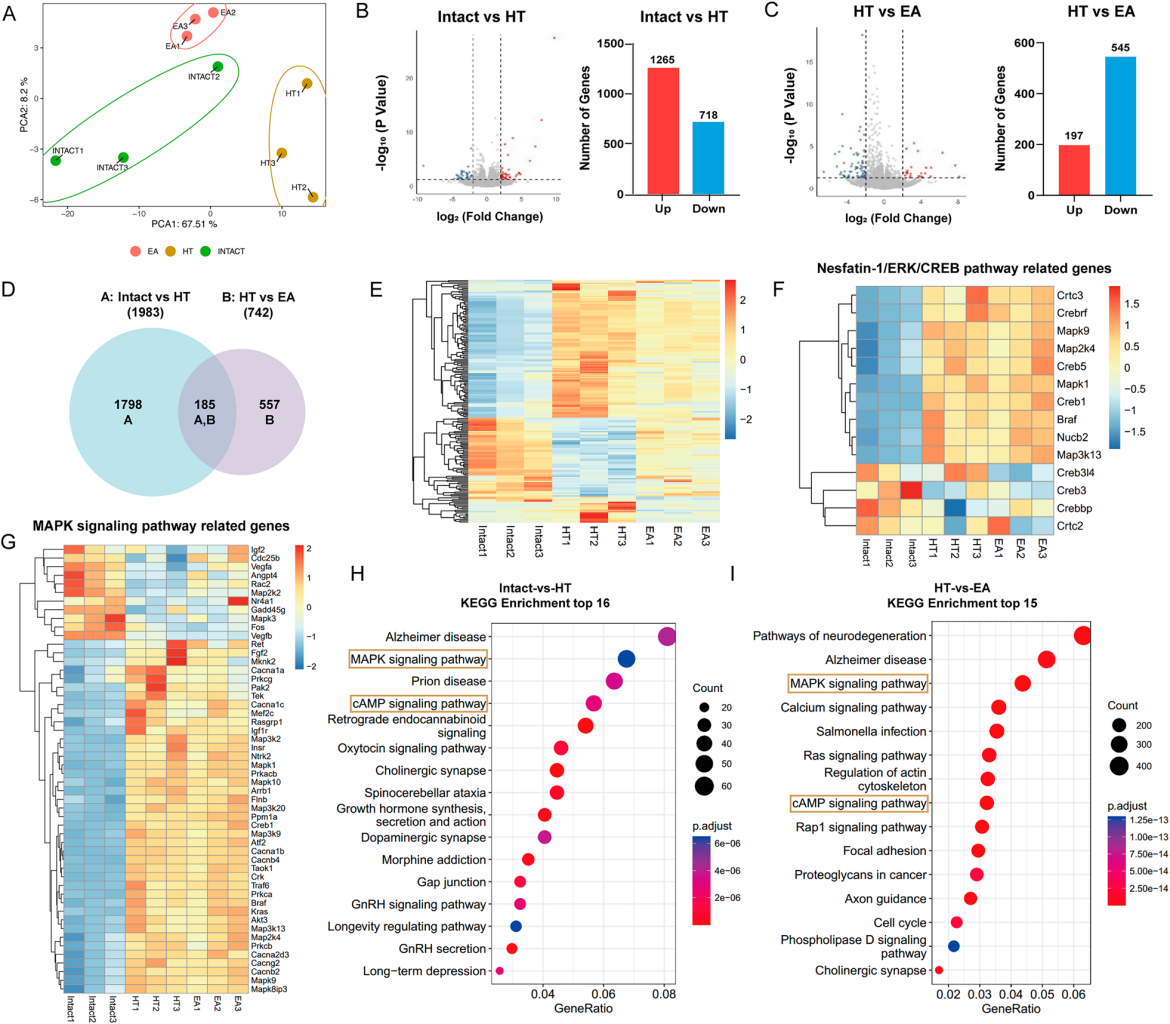
Depicts the distinct transcriptional profiles induced by surgical trauma and subsequent EA treatment. **A** PCA of the PVN. **B**, **C** Volcano plots illustrating the upregulated and downregulated genes after surgical trauma and subsequent EA treatment, showing the number of upregulated and downregulated genes in each condition (n = 3 mice per group; fold change > 1.5, adjusted p < 0.05). **D** Venn diagram showing the unique differentially expressed genes in the HT and EA + HT groups (n = 3 mice per group; fold change > 1.5, adjusted p < 0.05). **E** Heatmap of differentially expressed genes in the hypothalamus among the Control, HT, and EA + HT groups (n = 3 mice per group; fold change > 1.5, adjusted p < 0.05). **F**, **G** Heatmap of differentially expressed transcripts related to the Nesfatin-1/ERK/CREB pathway and MAPK pathway (n = 3 mice per group; fold change > 1.5, adjusted p < 0.05). **H**, **I** KEGG pathway analysis of differentially expressed genes (fold change > 1.5, adjusted p < 0.05) after surgical trauma and subsequent EA treatment

To investigate the potential mechanisms underlying surgical trauma-induced HPA axis hyperactivity, anxiety, and the therapeutic effects of EA, we conducted RNA sequencing on hypothalamic tissues 24 h after HT in rats. The PCA analysis suggested that the Intact, HT, and EA groups were distributed separately (Fig. 6A). The transcriptional profiles differed among the three groups, and samples within the same group clustered together (Fig. 6E). The results revealed 1983 differentially expressed genes (1265 upregulated, 718 downregulated) between the HT and Intact groups (Fig. 6B). For the HT + EA group compared to the HT group, there were 742 differentially expressed genes (197 upregulated, 545 downregulated) (Fig. 6C). Among these significantly changed genes, 557 were unique to the EA (Fig. 6D).

Subsequently, KEGG pathway enrichment analysis was performed on the differentially expressed genes (fold change > 1.5, adjusted p < 0.05). The analysis revealed that the mitogen-activated protein kinase (MAPK) and cAMP signaling pathways were regulated after surgical trauma and EA (Fig. 6H, I). These pathways could activate downstream extracellular ERK and CREB pathways. The significant upregulation of genes related to the Nesfatin-1/ERK/CREB pathway and the MAPK signaling pathway is consistent with previous studies [32, 33] (Fig. 6F, G). This suggests that the hypothalamic changes induced by surgical trauma and EA may be mediated through the ERK/CREB pathway.

### Nesfatin-1 regulates CRH expression by activating the ERK/CREB pathway

**Fig. 7:**
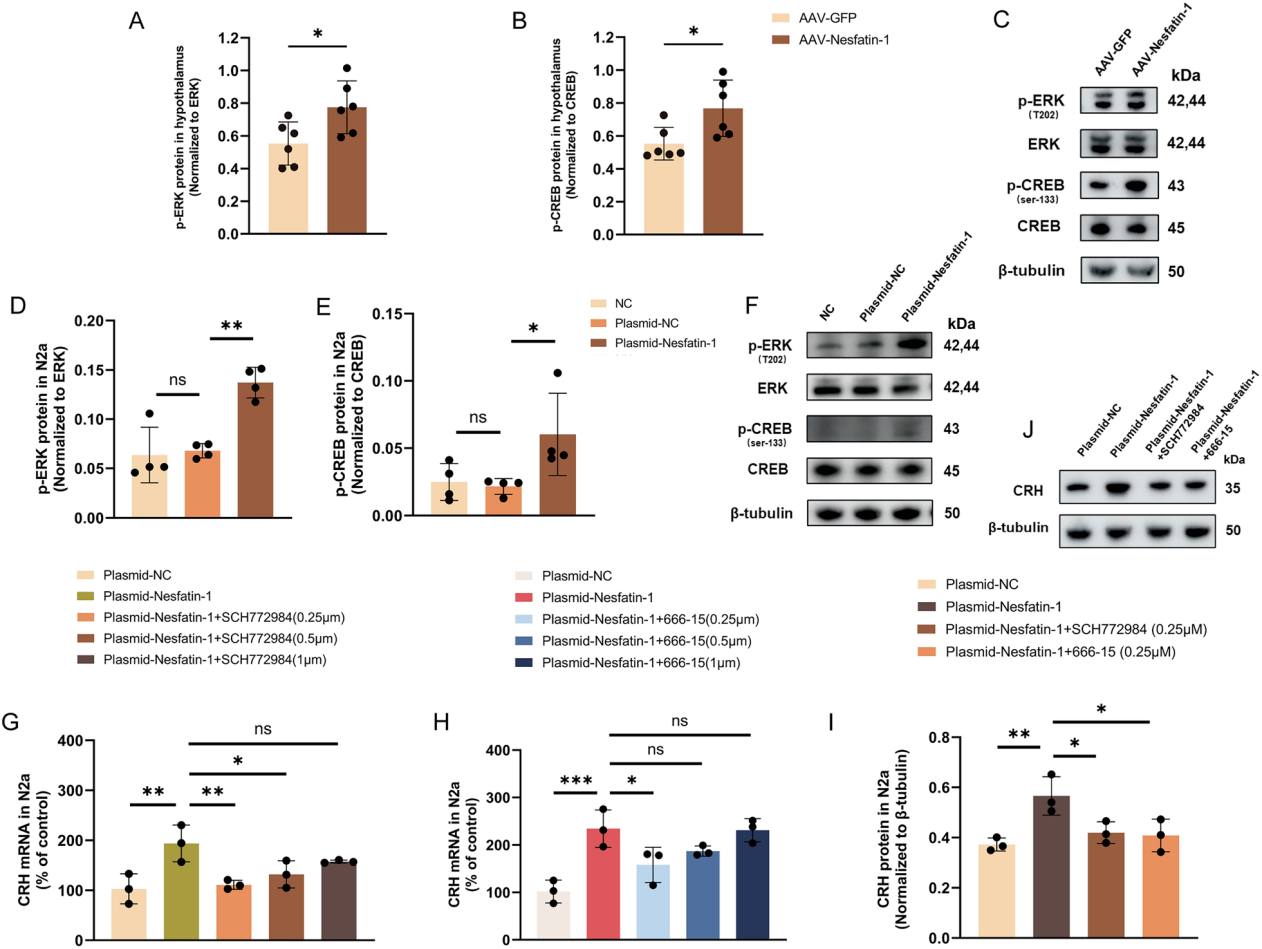
Nesfatin-1 regulates CRH expression by activating the ERK/CREB pathway. **A**–**C** The phosphorylation levels of ERK and CREB in hypothalamus from each group. **D**–**F** The phosphorylation levels of ERK and CREB in N2a cells from each group. **G**–**H** Levels of CRH mRNA in N2a cells from each group. **I**–**J** Levels of CRH protein in N2a cells from each group. All data are shown as mean ± SEM, n = 3–6 in each group, * p < 0.05, ** p < 0.01, ***p < 0.001

Based on the transcriptome sequencing results, we first investigated whether Nesfatin-1 could modulate the ERK/CREB pathway. We injected AAV-Nesfatin-1 into the PVN to achieve overexpression of Nesfatin-1 (Fig. 3A). Upon confirming successful overexpression of Nesfatin-1 (Fig. 3B–D), we observed a significant increase in phosphorylation levels of ERK and CREB in the hypothalamus of mice in the AAV-Nesfatin-1 group compared to the AAV-GFP group (Fig. 7A–C). Concurrently, we performed plasmid transfection of N2a cells to achieve overexpression of Nesfatin-1 (Fig. 5A). Following confirmation of successful overexpression of Nesfatin-1 (Fig. 5C, D), we observed a significant increase in phosphorylation levels of ERK and CREB in cells of the Nesfatin-1 overexpressing group compared to the control group (Fig. 7D–F). These results confirm the sufficiency of Nesfatin-1 in activating the ERK/CREB pathway.

To further investigate the role of the ERK/CREB pathway in Nesfatin-1-mediated CRH secretion, we treated N2a cells with different concentrations of ERK inhibitor (SCH772984) and CREB inhibitor (666–15) concurrently with Nesfatin-1 overexpression. We observed that compared to 0.5 μm and 1 μm concentrations, 0.25 μm of SCH772984 and 666–15 exhibited significantly stronger inhibitory effects on the elevation of CRH mRNA (Fig. 7G, H) and protein (Fig. 7I, J) induced by Nesfatin-1 overexpression. These results validate the necessity of the ERK/CREB pathway in mediating the increase in CRH induced by Nesfatin-1.

### Inhibition of ERK and CREB alleviates the HPA axis hyperactivity and anxiety-like behaviors caused by surgical trauma

**Fig. 8:**
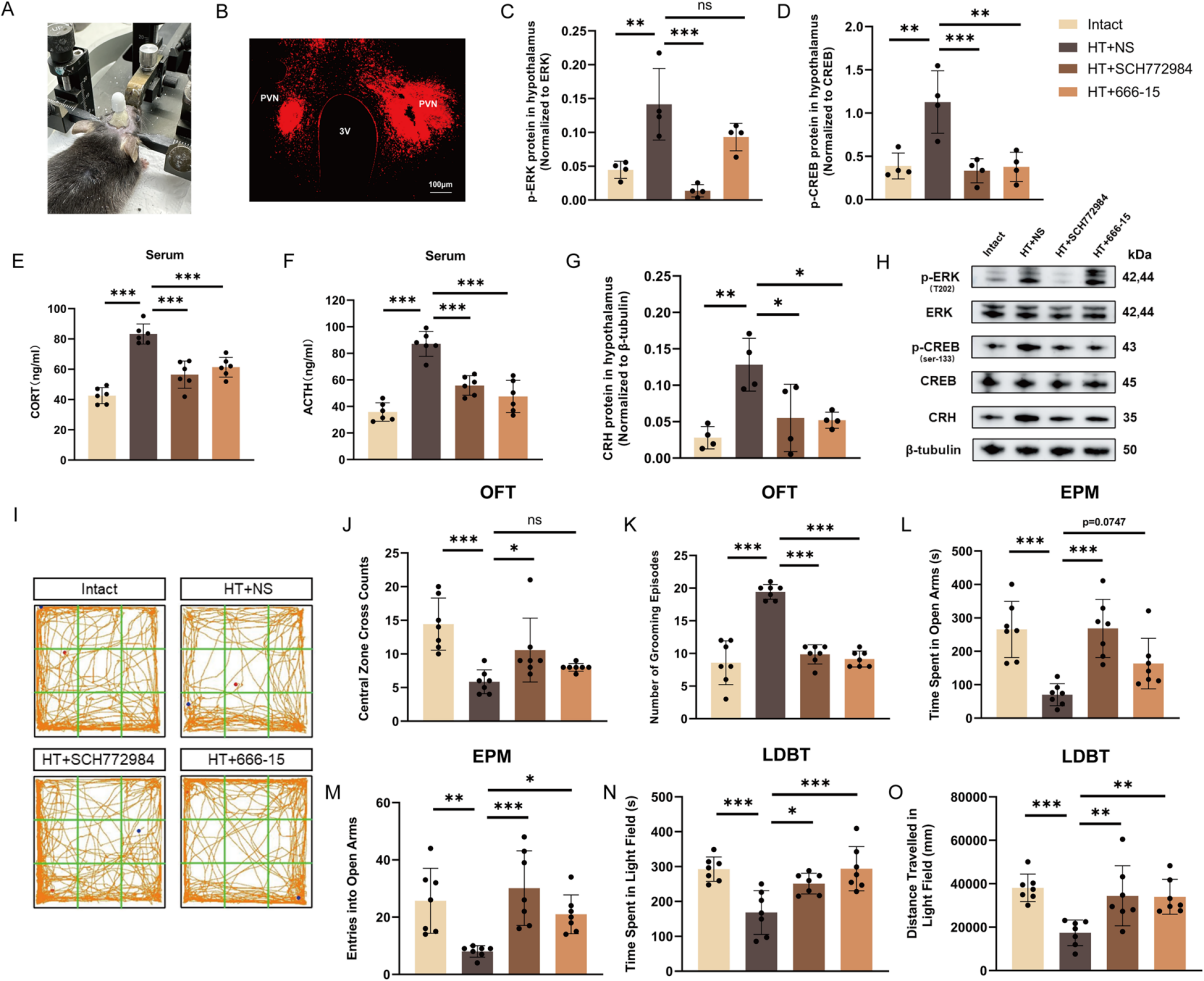
Inhibition of ERK and CREB alleviates the HPA axis hyperactivity and anxiety-like behaviors caused by surgical trauma. **A** Stereotactic cannula implantation. **B** Diagram illustrating the sites of drug administration. Scale bar = 100 μm. **C**, **D, G**, **H** The phosphorylation levels of ERK and CREB and CRH protein level in hypothalamus from each group. **E**, **F** Serum levels of ACTH and CORT in different groups. OFT (**I**–**K**), EPM (**L**, **M**), and LDBT (**N**, **O**) were used to assess anxiety levels in different groups of mice. All data are shown as mean ± SEM, n = 4–7 in each group, * p < 0.05, ** p < 0.01, ***p < 0.001

We administered 0.25 μm of SCH772984, 666–15 and NS into the PVN (Fig. 8A, B). The results showed that SCH772984 significantly decreased the phosphorylation levels of ERK and CREB in the hypothalamus of HT + NS mice (Fig. 8C, D), while 666–15 significantly reduced the phosphorylation level of CREB in the hypothalamus of these mice (Fig. 8D). Both drugs significantly reverse the increase of serum CORT, ACTH, and hypothalamic CRH protein levels induced by surgical trauma (Fig. 8E–H). These results indicate that activation of the hypothalamic ERK/CREB pathway is necessary for the hyperactivity of the HPA axis in mice subjected to surgical trauma.

Furthermore, we assessed behavioral indices in mice after administration of the inhibitors. OFT results revealed that both inhibitors significantly reversed the decrease in central zone cross counts and the increase of grooming episodes in HT + NS mice (Fig. 8I–K). EPM results indicated that both inhibitors significantly reversed the decrease in time spent in the open arms and entries into the open arms in HT + NS mice (Fig. 8L, M). LDBT results demonstrated that both inhibitors significantly reversed the decrease in time spent and distance traveled in the light area in HT + NS mice (Fig. 8N, O). These results suggest that activation of the hypothalamic ERK/CREB pathway is necessary for anxiety-like behaviors in mice subjected to surgical trauma.

### EA inhibits surgical trauma-induced activation of the hypothalamic ERK/CREB pathway

**Fig. 9:**
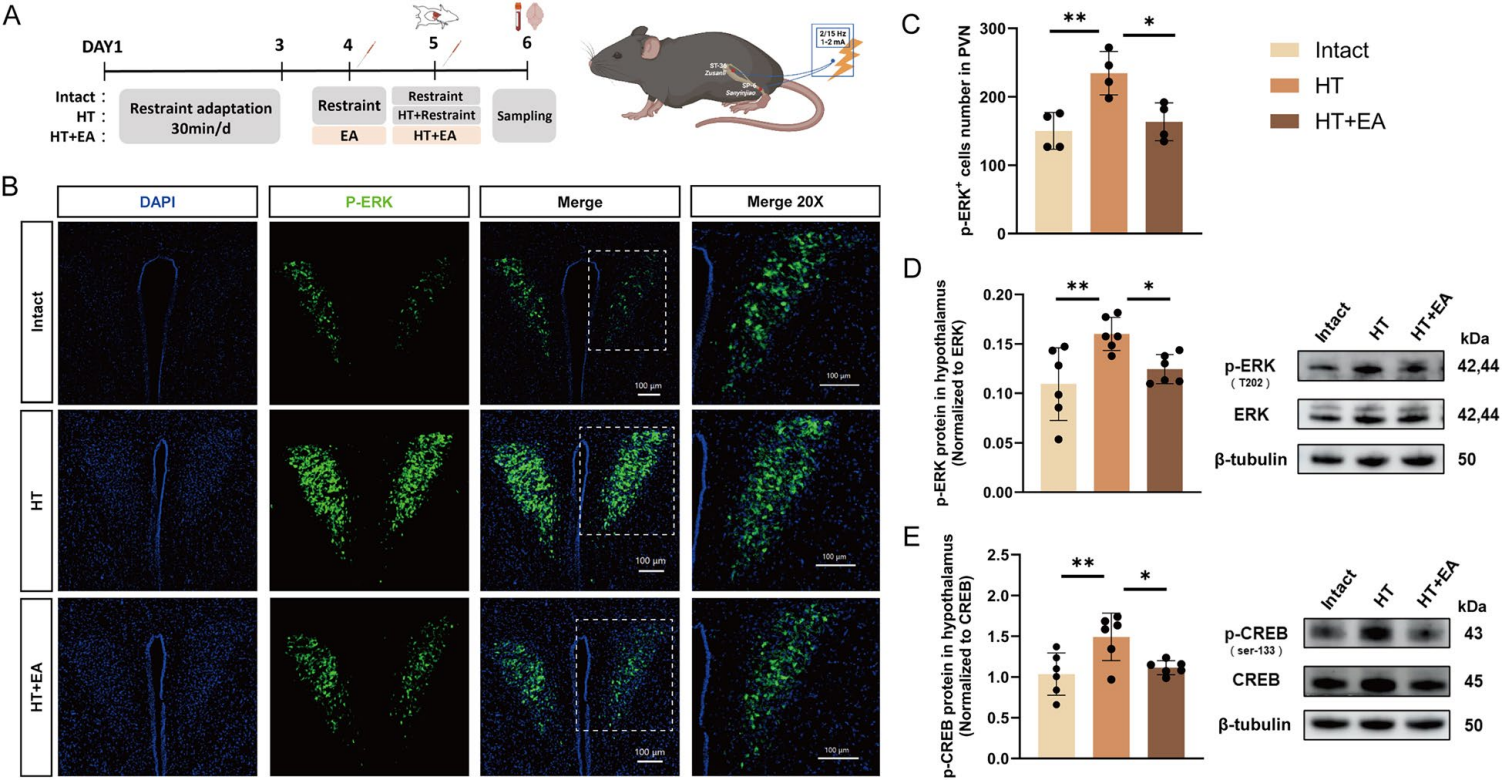
EA alleviates the activation of the hypothalamic ERK/CREB pathway induced by surgical trauma. **A** Schematic representation of the HT model and EA protocol. **B** Immunofluorescent staining of p-ERK and DAPI in the PVN of mice from the Intact, HT, and EA + HT groups 24 h post-surgery. Scale bar = 100 μm. **C** Quantification of p-ERK-positive cells in each group 24 h post-surgery. **D** Phosphorylation levels of ERK in the hypothalamus in each group. **E** Phosphorylation levels of CREB in the hypothalamus in each group. All data are shown as mean ± SEM, n = 4–6 in each group, * p < 0.05, ** p < 0.01, ***p < 0.001

We examined the changes in the hypothalamic ERK/CREB signaling pathway in mice subjected to surgical trauma and subsequent EA. The experimental workflow is depicted in Fig. 9A. Compared to the Intact mice, the HT mice exhibited a significant increase in the number of PVN p-ERK-positive cells, as well as elevated phosphorylation levels of hypothalamic ERK and CREB (Fig. 9B–E). Importantly, these increases were effectively suppressed by EA (Fig. 9B–E). This confirms that the activation of the hypothalamic ERK/CREB pathway induced by surgical trauma could be inhibited by EA.

## Discussion

This study clarifies the role of EA in reducing hyperactivity of the HPA axis and consequent anxiety triggered by acute surgical trauma. Utilizing virological tools and a combination of in vitro and in vivo experiments, we have conclusively established that Nesfatin-1 plays a crucial role in mediating the effects of EA on both endocrine and emotional disturbances. Moreover, we have identified ERK and CREB as key molecules in the Nesfatin-1-mediated regulation of CRH. The Nesfatin-1/ERK/CREB pathway is effectively downregulated by the EA treatment, leading to the alleviation of trauma-induced HPA axis hyperactivity and subsequent anxiety.

The HPA axis plays a crucial role in regulating homeostasis and responses to threats from both internal and external environments. However, excessive activation of the HPA axis often leads to a series of harmful effects and adverse reactions [34]. Surgical trauma, as a form of acute stress, can lead to HPA axis hyperactivity. Partial HT induces tissue ischemia, hypoxia, as well as abnormalities in endocrine, metabolic, and immune functions, providing a robust simulation of surgical stress response [35]. In this study we employed a partial HT model to simulate surgical trauma. The impact of HT model on the HPA axis function has been preliminarily explored. Our previous studies indicated varying degrees of HPA axis hyperactivity at 2 h, 4 h, 6 h, 24 h, and 72 h after HT in rats [35, 36]. Hyperactivity of HPA axis can result in endocrine disruption and serve as the primary initiating factor for severe secondary injuries such as systemic inflammatory response syndrome and multiple organ dysfunction syndrome [37]. CRH, secreted by parvocellular neurons in the PVN, acts as the “gatekeeper” for initiating HPA axis and is a significant factor contributing to anxiety [38, 39]. Studies have shown elevated central CRH levels in stress-related anxiety and depressive patients, with normalization observed after treatment [40]. Corticotropin releasing hormone receptor 1 (CRHR1) antagonists such as antalarmin [41] and miR-34b, which targets CRHR1 and negatively regulates its mRNA [30], have been reported to alleviate anxiety. Additionally, anxiety patients exhibit evident HPA axis hyperactivity, and chronic activation of the HPA axis has been linked to the development and exacerbation of anxiety [42]. Therefore, the excessive secretion of PVN CRH, causing HPA axis hyperactivity, deserves attention due to its role in inducing anxiety and adverse consequences following surgical trauma. Here, we observed HPA axis activation after surgery, evidenced by elevated PVN CRH levels and increased serum ACTH and CORT levels. Similarly, through behavioral experiments, mice exhibited pronounced anxiety-like behaviors, consistent with previous experimental results [30].

A significant body of research indicates that EA has a favorable anti-stress effect by regulating the HPA axis. In various conditions such as anxiety and depression [43], cardiovascular diseases [10], immune suppression [44], irritable bowel syndrome [45] and diabetes [46], the use of EA has been shown to alleviate HPA axis hyperactivity, achieving therapeutic benefits. During the perioperative period, the application of EA can alleviate preoperative anxiety and tension in patients, reduce the use of anesthetics during surgery [47], and effectively relieve postoperative pain and gastrointestinal discomfort [12, 48]. Studies have shown that preoperative acupuncture is an effective intervention to alleviate patient anxiety [13]. Additionally, EA before gynecological laparoscopic surgery can improve postoperative analgesia and reduce postoperative side effects [49]. The selection of acupoints is a crucial factor influencing the efficacy of EA. ST36 and SP6 are the most commonly used acupoint combinations in clinical practice [50]. Studies indicate that EA at ST36 can alleviate anxiety and depression levels in rats subjected to unpredictable chronic mild stress (UCMS) by regulating the HPA axis [51]. Meanwhile, our previous research indicated that non-acupoint intervention (where acupuncture needles are inserted into the ipsilateral tail root and electrical stimulation is applied) failed to improve HPA axis dysfunction in traumatized animals [52]. Our research confirms that preoperative EA pretreatment combined with postoperative EA can effectively alleviate HPA axis hyperactivity and anxiety.

Although the beneficial regulatory effects of EA on the HPA axis have been widely confirmed, its specific mechanisms remain unclear. Previous studies have indicated that EA can enhance the inhibitory regulation of the hypothalamic gamma-aminobutyric acid-A receptor α3 subunit [18], inhibit the phosphorylation of hypothalamic N-methyl-D-aspartate receptor 2A [29], thereby reducing excessive secretion of CRH, and alleviate HPA axis hyperactivity induced by surgical trauma. Recent research also suggests that EA can reverse the downregulation of hypothalamic oxytocin and oxytocin receptor induced by surgical trauma, downregulate glucocorticoid receptor expression [17], and impact the circRNA-miRNA-mRNA network [19], suppressing HPA axis hyperactivity. In this study, we investigated the role of a novel neuropeptide, Nesfatin-1, in surgical trauma. Nesfatin-1, first discovered in the hypothalamus in 2006, is derived from NUCB2 [53] and consists of three domains: N-terminal, middle portion (M30), and C-terminal, with M30 playing a crucial role in inducing physiological effects [54]. Previous studies have indicated that Nesfatin-1 plays a significant role in stress regulation. Co-localization of Nesfatin-1 and CRH in the PVN has been observed [55]. Nesfatin-1 neurons in multiple stress-related brain regions, including the PVN, are significantly activated in various stress conditions such as restraint stress [56], water avoidance stress [57], abdominal surgery [58], and lipopolysaccharide injection [59]. Three weeks of UCMS in rats leads to a significant increase in Nesfatin-1 and CRH mRNA levels in the hypothalamus, as well as elevated serum CORT levels [60]. Central injection of Nesfatin-1 induces enhanced HPA axis and sympathetic nervous system activity [61], anxiety-like behaviors [26, 62], and changes in visceral function [63]. In human studies, high levels of Nesfatin-1 have been detected in the plasma of individuals diagnosed with severe depression [64], and serum Nesfatin-1 in obese women correlates positively with perceived stress, anxiety, and depression levels [65]. These findings suggest the involvement of Nesfatin-1 in the regulation of stress and stress-related psychiatric disorders. Our research results showed that in HT mice, Nesfatin-1 expression in the hypothalamus significantly increased, and EA reduced its elevated expression. Furthermore, stereotactic injection of Nesfatin-1 overexpression virus into the PVN could simulate HPA axis hyperactivity and anxiety-like behaviors induced by surgical trauma, while Nesfatin-1 knockdown virus could inhibit HPA axis hyperactivity and anxiety-like behaviors induced by surgical trauma, like the effects of EA. This indicates that EA has the potential to alleviate HPA axis hyperactivity and anxiety after surgical trauma by inhibiting the elevated levels of endogenous Nesfatin-1 in the hypothalamus.

Although the receptors for Nesfatin-1 have not been definitively identified, the intracellular signaling pathways induced by Nesfatin-1 have been extensively studied [66]. In our experiment, we clarified the positive regulatory effect of Nesfatin-1 on CRH in N2a cells and used transcriptome sequencing to explore the downstream signaling pathways mediated by Nesfatin-1. ERK is a member of the MAPK family and plays a crucial role in transmitting surface receptor signals to the cell nucleus [67]. CREB is a cAMP response element-binding protein that selectively binds to cAMP response elements (CRE), regulating the transcription of various cellular genes. Transcriptome sequencing revealed that genes significantly altered by HT and EA were enriched in the MAPK and cAMP signaling pathways, both of which activated downstream ERK and CREB. In the PVN, ERK could phosphorylate CREB and promote its translocation to the nucleus, where it binds with CRE to activate the transcription of CRH [68]. Studies have shown that Nesfatin-1 treatment of SH-SY5Y cells increases the phosphorylation of ERK1/2 and CRH levels [69]. Microinjection of Nesfatin-1 into the PVN of rats increases the number of p-ERK1/2-positive cells and CRH levels [70]. Treatment of NB41A3 cells with Nesfatin-1 or M30 increases the phosphorylation levels of CREB [71]. Our research results show that overexpression of Nesfatin-1 in N2a cells could activate the ERK/CREB pathway, and application of ERK or CREB inhibitors could reverse the increase in CRH caused by Nesfatin-1 overexpression. EA, on other hand inhibits the activation of the hypothalamic ERK/CREB pathway induced by surgical trauma. Microinjection of ERK or CREB inhibitors into the PVN of mice can alleviate HPA axis hyperactivity and anxiety-like behaviors caused by surgical trauma, similar to the effects of EA. This suggests that the Nesfatin-1/ERK/CREB pathway is involved in HPA axis hyperactivity and anxiety caused by surgical trauma and can be inhibited by EA.

The HPA axis undergoes dynamic changes and exhibits a circadian rhythm. In this study, we only explored the time point of 24 h after surgery, which may not provide a comprehensive understanding. Additionally, although mice showed good recovery 24 h postoperatively, the abdominal incision is likely to affect their motor function. Therefore, we corrected the OFT data, focusing on the ratio of the distance moved in the central area to the total distance moved and the speed during movement, rather than average speed. It is worth noting that these indicators were significantly improved after EA treatment, which may extend beyond the regulatory effect of EA on the HPA axis, involving overall regulation, including anti-inflammatory and analgesic effects, aligning with traditional Chinese medicine principles. Furthermore, gender differences were not considered in this study. Since research has indicated that expression of Nesfatin-1 seems to be higher in the brains of males than females with depression [55]. However, our experiment only investigated male mice. These limitations should be thoroughly addressed in future research endeavors.

## Conclusion

**Fig. 10:**
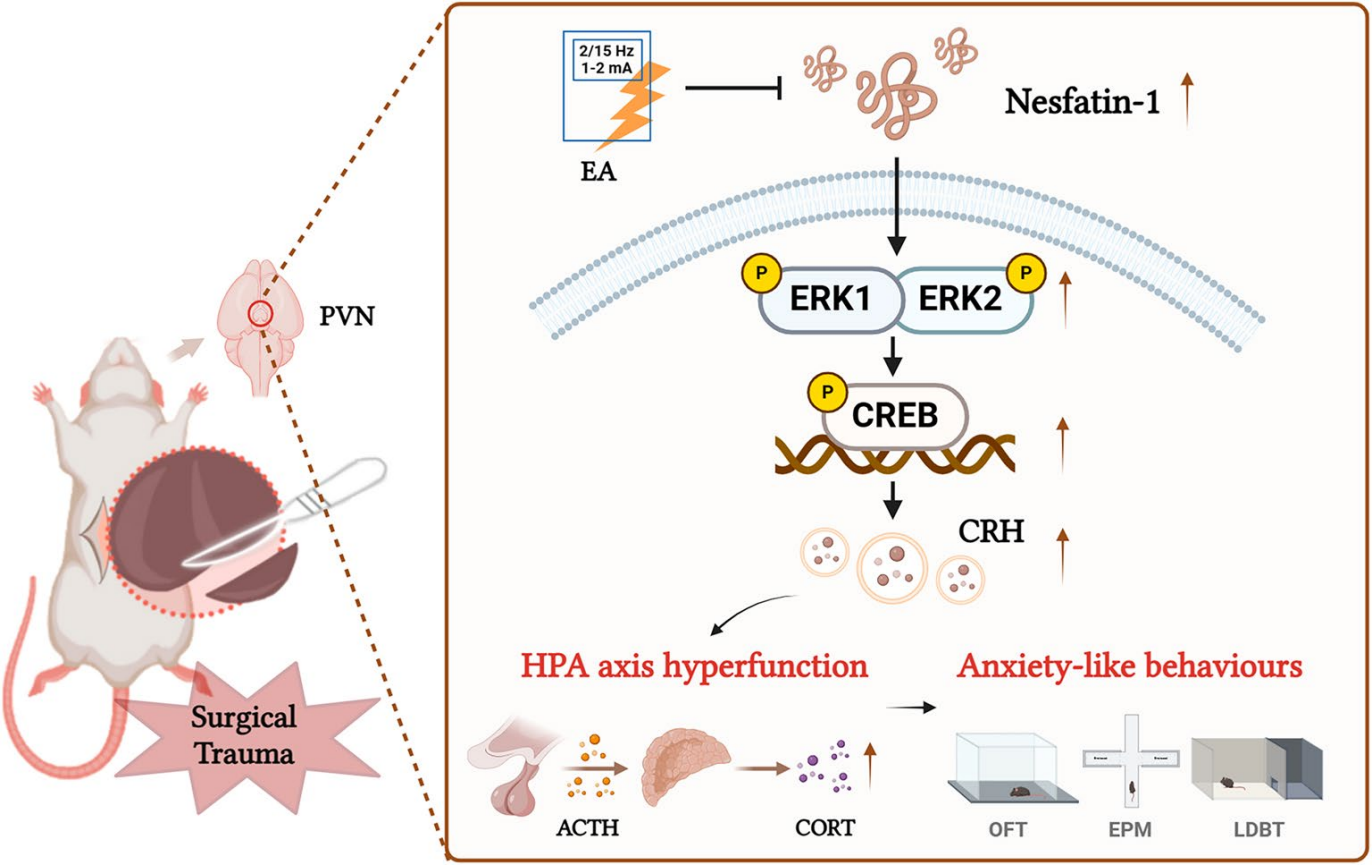
Electroacupuncture modulates Nesfatin-1/ERK/CREB pathway to reduce surgical trauma-induced HPA axis hyperactivity and anxiety. Figure created with BioRender.com

In conclusion, our study demonstrates that the hypothalamic Nesfatin-1/ERK/CREB pathway may be involved in the inhibitory effects of EA on surgical trauma-induced HPA axis hyperactivity and anxiety (Fig. 10).

## Supplementary Information

Supplementary Material 1.

## Data Availability

The datasets used or analyzed throughout this study are available from the corresponding author upon reasonable request.

## Abbreviations

ACTH: Adrenocorticotropic hormone
CRE: CAMP response elements
CRH: Corticotropin-releasing hormone
CRHR1: Corticotropin releasing hormone receptor 1
CORT: Corticosterone
CREB: CAMP-response element binding protein
EA: Electroacupuncture
EPM: Elevated plus maze
ERK: Extracellular regulated protein kinases
HPA: Hypothalamic–pituitary–adrenal
HT: Hepatectomy
LDBT: Light–dark box tests
MAPK: Mitogen-activated protein kinase
M30: Middle portion
NS: Normal saline
NUCB2: Nucleobindin2
N2a: Neuroblastoma-2a
OFT: Open field tests
PAC: Principal component analysis
PVN: Paraventricular nucleus
SD: Sprague–Dawley
UCMS: Unpredictable chronic mild stress
3V: Third ventricle
