# Correction: Electroacupuncture negatively regulates the Nesfatin-1/ERK/CREB pathway to alleviate HPA axis hyperactivity and anxiety-like behaviors caused by surgical trauma

**DOI:** 10.1186/s13020-024-00999-7

**Published:** 2024-09-29

**Authors:** Jiayuan Zheng, Yu Wang, Chi Zhang, Anjing Zhang, Yuxiang Zhou, Yunhua Xu, Jin Yu, Zhanzhuang Tian

**Affiliations:** 1 Department of Integrative Medicine and Neurobiology, School of Basic Medical Sciences, State Key Laboratory of Medical Neurobiology and MOE Frontiers Center for Brain Science, Institutes of Brain Science, Institute of Acupuncture Research, Academy of Integrative Medicine, Shanghai Key Laboratory for Acupuncture Mechanism and Acupoint Function, Shanghai Medical College, Fudan University, Shanghai, 200032 China; 2 Department of Medical Oncology, Zhongshan Hospital, Fudan University, Shanghai, 200032 China; 3 Department of Rehabilitation Medicine, Huashan Hospital, Fudan University, Shanghai, 200040 China; 4 Department of Neurological Rehabilitation Medicine, The First Rehabilitation Hospital of Shanghai, Shanghai, 200090 China

**Correction: Chinese Medicine (2024) 19:108** 10.1186/s13020-024-00974-2

Following publication of the original article [1], the authors reported inaccuracies in the sub-images of Fig. 5 and the Funding section. They have corrected these errors with the accurate sub-images from the correct version. These corrections do not alter the results or conclusions of their study.

The correct Fig. 5 and Funding are provided in this Correction.

The incorrect Fig. 5 is:

**Fig. 5:**
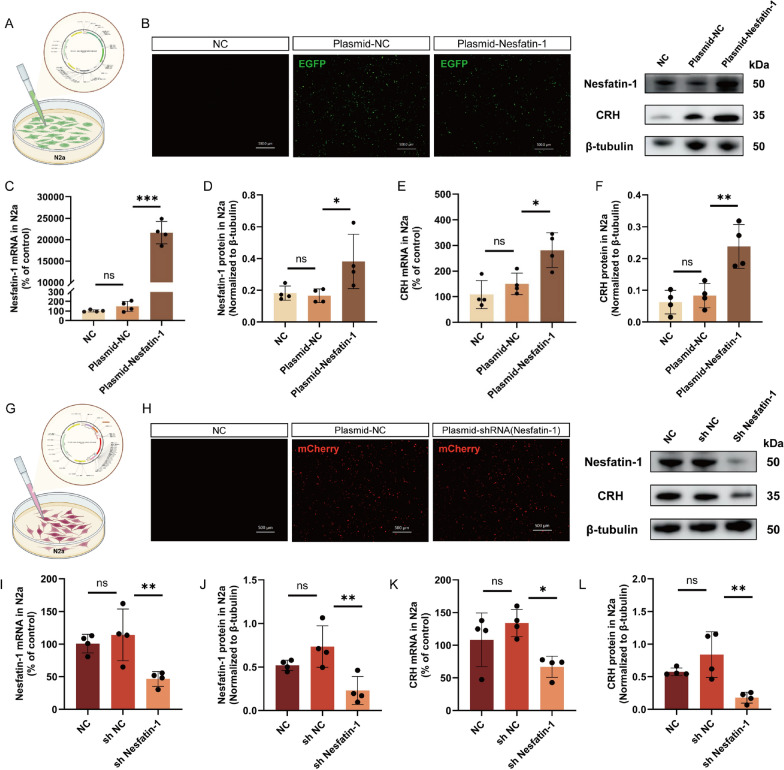
Fig. 5 Regulation of CRH expression by Nesfatin-1. **A**, **G** Schematic representation of plasmid transfection. **B**, **H** Fluorescent images of N2a cells after 48 h of plasmid transfection. Scale bar = 500 μm. **C**,** D**,** I**,** J** Expression levels of Nesfatin-1 mRNA and protein in cells from each group. **E**,** F**, **K**,** L** Expression levels of CRH mRNA and protein in cells from each group. All data are shown as mean ± SEM, n = 4 in each group, * p < 0.05, ** p < 0.01, ***p < 0.001

The correct Fig. 5 is:

**Fig. 5:**
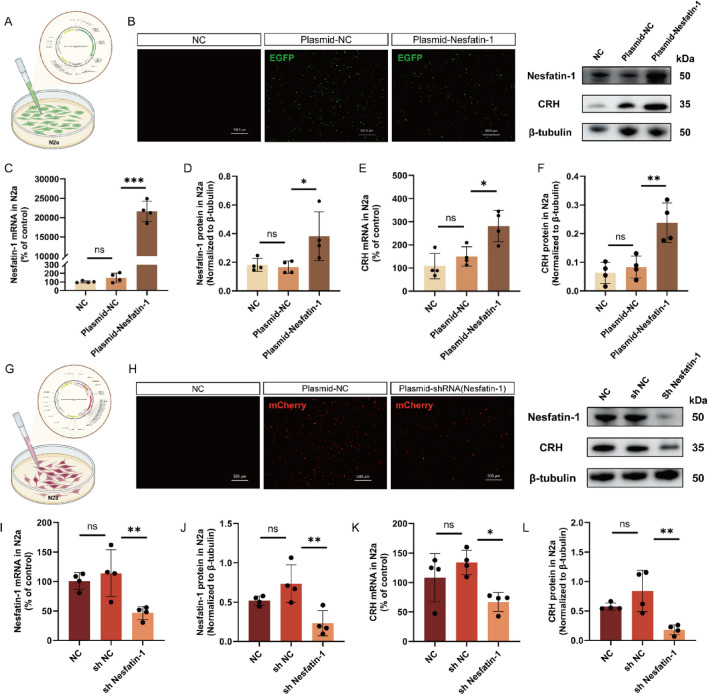
Regulation of CRH expression by Nesfatin-1. **A**, **G** Schematic representation of plasmid transfection. **B**, **H** Fluorescent images of N2a cells after 48 h of plasmid transfection. Scale bar = 500 μm. **C**,** D**,** I**,** J** Expression levels of Nesfatin-1 mRNA and protein in cells from each group. **E**,** F**, **K**,** L** Expression levels of CRH mRNA and protein in cells from each group. All data are shown as mean ± SEM, n = 4 in each group, * p < 0.05, ** p < 0.01, ***p < 0.001

The incorrect Funding is:

This work was supported by the National Natural Science Foundation of China (Grants numbers 81973639 and 81573712) and the Innovative Research Team of High-level Local Universities in Shanghai.

The correct Funding is:

This work was supported by the National Natural Science Foundation of China (Grants numbers 82474210, 81973639 and 81573712) and the Innovative Research Team of High-level Local Universities in Shanghai.

The original article [1] has been corrected.

## References

[1] Zheng J, Wang Y, Zhang C, et al. Electroacupuncture negatively regulates the Nesfatin-1/ERK/CREB pathway to alleviate HPA axis hyperactivity and anxiety-like behaviors caused by surgical trauma. Chin Med. 2024;19:108. 10.1186/s13020-024-00974-2. 39153974 10.1186/s13020-024-00974-2 PMC11330601

